# Editorial: Machine-learning/deep-learning methods in neuromarketing and consumer neuroscience

**DOI:** 10.3389/fnhum.2025.1638225

**Published:** 2025-06-23

**Authors:** Marco Bilucaglia, Luca Mainardi, Thomas Zoëga Ramsøy, Paul J. Zak, Margherita Zito, Vincenzo Russo

**Affiliations:** ^1^Department of Business, Law, Economics and Consumer Behaviour “Carlo A. Ricciardi”, Università IULM, Milan, Italy; ^2^Behavior and Brain Lab IULM – Neuromarketing Research Centre, Università IULM, Milan, Italy; ^3^SpinLabs, Dipartimento di Elettronica, Informazione e Bioingegneria, Politecnico di Milano, Milan, Italy; ^4^Neurons Inc., Høje Taastrup, Denmark; ^5^International Center for Applied Neuroscience, Rørvig, Denmark; ^6^Center for Neuroeconomics Studies, Claremont Graduate University, Claremont, CA, United States; ^7^Immersion Neuroscience, Henderson, NV, United States

**Keywords:** consumer neuroscience and neuromarketing, machine learning, deep learning, consumer behavior, XAI and explainable artificial intelligence, artificial intelligence (AI), consumer behavior and decision-making theory, neuroscience methods

This Research Topic explores the application of Machine Learning (ML) and Deep Learning (DL) methods in Neuromarketing and Consumer Neuroscience. Rather than following the prevailing “AI trend” (Messeri and Crockett, 2024), we aim to promote data-driven approaches as effective solutions to two persistent challenges in the field: low ecological validity and the issue of reverse inference (Yao and Wang, 2024). These challenges arise when mental states (*m*) are inferred from neurophysiological indices (*x*) that, although grounded in neuroimaging research, are often collected under artificial laboratory conditions. Their correlational nature (*x*|*m*) also undermines their predictive utility (*m*|*x*) unless both the prior (P(m)) and base rate (P(x)) are properly specified (Plassmann et al., 2015; Ramsøy, 2019).

ML/DL methods address these issues in two ways. First, they estimate P(m|x), facilitating a more robust variant of reverse inference known as “pattern-based decoding” (Nathan and Pinal, 2017). Second, when trained in ecologically valid contexts (e.g., consumer decision-making or exposure to marketing stimuli), they enhance generalizability without sacrificing accuracy (Shamay-Tsoory and Mendelsohn, 2019). Free from predefined assumptions, these methods can also uncover latent structures in physiological data, offering new theoretical insights and hypotheses (Verzelli et al., 2024).

Despite these advantages, their adoption remains limited (Song et al., 2025). To date, only 35% of studies in Neuromarketing and Consumer Neuroscience (20 of 57) have utilized ML/DL methods (Rawnaque et al., 2020), with this percentage varying between 27% (23 of 86) (Byrne et al., 2022) and 50% (43 of 86) (Khondakar et al., 2024) in EEG-focused studies.

This Research Topic features eight contributions: six experimental studies, one dataset paper, and one meta-analysis. The investigated mental states range from emotion and engagement to preference and willingness to pay. Classifiers include both traditional and ensemble models, as well as shallow and deep neural networks. Modalities include the electrical and metabolic activity of both the peripheral and central nervous systems. Below is a brief overview of the contributions, presented in order of publication.

Hakim et al. explored willingness to pay (WTP) using EEG recordings from 231 participants while viewing 72 product images. A deep architecture combining CNN and RNN with attention layers was trained on raw signals, achieving 75.09% accuracy in binary classification and a 0.276 RMSE in continuous prediction. Filter-based model explainability highlighted beta, delta, and alpha bands, as well as the frontal region (FPz) as the most predictive features.

Merritt et al. predicted hit songs based on peripheral measures of “immersion” and “retreat” in 33 participants exposed to 24 tracks (13 of which were hits). A logistic regression model reached 69% accuracy, which was improved to 97% by a stacked ensemble of kNN, SVM, and ANN. Using only the first 60 seconds of data, the model retained 82% accuracy.

Çakar and Filiz examined political engagement using fNIRS in 33 participants rating 12 political leader images accompanied by valenced descriptors. A range of models was tested, with LightGBM achieving the best accuracy of 78%. SHAP analysis identified dorsomedial and ventromedial prefrontal activations as key predictors of political preference.

Polo et al. investigated emotional responses to visual, auditory, and bimodal stimuli through EEG, SC, PPG, respiration, and pupillometry in a 22-participants sample. Standardized emotional content covered all arousal-valence quadrants. Among several tested classifiers, AdaBoost performed best (52% accuracy for auditory, 44% for visual, and 51% for combined). The proposed novel Square Method feature selection identified EEG, SC, ECG, and respiration as the most informative modalities.

Watanuki studied consumer decisions involving branded vs. unbranded foods by analyzing 32 previously published fMRI studies (2000–2023) using Activation Likelihood Estimation and Multi-Coordinate Pattern Analysis via sparse Partial Least Squares Discriminant Analysis. The ventromedial prefrontal cortex was consistently activated in both conditions, while the lingual and parahippocampal gyri were uniquely responsive to branded stimuli. The latter also emerged as a key region for discriminating brand engagement.

Çakar et al. addressed credit decision-making using fNIRS recorded while 39 participants evaluated 35 loan offers. Features were input to several models, with Extra Trees achieving the highest accuracy of 79%. SHAP analysis related the prediction outcomes to activation in dorsolateral, orbitofrontal and ventromedial prefrontal regions.

Bilucaglia et al. presented I DARE, a multimodal dataset of physiological responses obtained from 63 participants exposed to 32 emotional images. Stimuli were drawn from standard affective databases and selected semi-automatically based on valence-arousal geometry and raters' agreement. The dataset, that includes pre-processed EEG, SC, PPG, EMG and eye-tracking data, is available at https://figshare.com/projects/I_DARE/186558.

Finally, Marques dos Santos and Marques dos Santos examined brand perception using fMRI data from 22 participants shown 160 stimuli. A MLP was trained on BOLD responses achieved 55.9% accuracy. SHAP and path-weight analysis identified early visual areas, particularly the cuneal and lateral occipital cortices, as key contributors to preference discrimination.
